# Identification of potential inhibitors for Penicillinbinding protein (PBP) from Staphylococcus aureus

**DOI:** 10.6026/97320630014471

**Published:** 2018-11-02

**Authors:** Langeswaran Kulanthaivel, Jeyakanthan Jeyaraman, Abir Biswas, Gowtham Kumar Subbaraj, S Santhoshkumar

**Affiliations:** 1 Cancer Genetics and Molecular Biology Laboratory, Department of Bioinformatics, Science Campus, Alagappa University, Karaikudi, Tamil Nadu, India; 2 Structural Biology and Bio-computing, Department of Bioinformatics, Science Campus, Alagappa University, Karaikudi, Tamil Nadu, India; 3 Abir Biswas, Molecular Gerontology Lab, Department of Biochemistry, Bharathidasan University,Thiruchirapalli, Tamil Nadu, India; 4 Faculty of Allied Health Sciences, Chettinad Hospital and Research Institute, Chettinad Academy of Research and Education, Kelambakkam, Chennai, Tamil Nadu, India; 5 Department of Computer Science, Alagappa University, Karaikudi, Tamil Nadu, India

**Keywords:** *Staphylococcus aureus*, penicillin binding protein, virtual screening, molecular docking

## Abstract

*Staphylococcus aureus* is an infectious agent that causes severe skin and soft tissue infection in hospitalized patients. Therefore, it is of interest to develop potent inhibitors for S. aureus. Penicillin Binding protein (PBP) is a known drug target for inhibition of cell wall biosynthesis in S. aureus. Hence, PBP was screened with compounds from six databases using virtual screening approaches. Results shows that the screened lead compound produced higher docking score (-9.87 kcal/mol) compared to resistant drugs. Antimicrobial activity using screened lead compounds and resistant drugs showed maximum activity in potential screened compounds compared to resistant compounds.

## Background

S. aureus is an antibiotic resistant pathogen that causes frequent serious infections 1. The penicillin binding proteins (PBPs) drug was commonly used as primary treatment for S. aureus for more than 40 years. Since 1980s, Centers for Disease Control and Prevention (CDC) was not recommending methicillin antibiotics because of its susceptibility and resistance against S. aureus 2. But current recommendation from the CDC for the treatment of S. aureus is with therapeutic methicillin, nafcillin, oxacillin, cloxacillin, dicloxacillin and flucloxacillin 3. Penicillin binding proteins (PBPs) are involved in the end stages of the synthesis of peptidoglycan, which is key component of bacterial cell wall. The inhibition of PBPs leads to irregularity in the form of bacterial cell wall structure such as elongation, lesions, the loss of permeability, and cell lysis 4. PBPs catalyze the synthesis of cross-linked individual peptidoglycan from lipid intermediates and the removal of D-alanine from the precursor of peptidoglycan. The purified enzymes showed following reaction such as D-alanine carboxypeptidase, peptidoglycan transpetidase and endo-peptidase activity in vitro. The N-terminal domain has penicillin-insensitive transglycosylase activity which is involved in the formation of linear glycan strands and Cterminal domain has penicillin-sensitive transpeptidase activity which is involved in the cross-linking peptide subunits, the active site serine was conserved in PBPs family 5.

## Methodology

### Protein Preparation:

The three-dimensional structure of penicillin binding protein was retrieved from the Protein Data Bank (PDB code: 1TVF). To perform the docking studies the protein structure was prepared by using protein preparation wizard available in schodinger 6. There are two steps involved in protein preparation. First one is the preparation, in this step the hydrogen was added and side chain atom was neutralized neither close to binding cavity nor involve in formation of salt bridges. Second step is refinement, in this step the water molecules were removed and h atoms were added and then it was minimized until it reaches the average root mean square deviation of the non-hydrogen atoms reached 0.3 Å.

### Ligand Preparation:

Structure of the ligands were retrieved from different chemical database namely Specs Databases, Enamics Databases, Maybridge Database and ZINC database. All the ligands were prepared were prepared using LigPrep module of Schrodinger 7. In the beginning, hydrogen atom was added and then the most relevant ionization and tautomeric states were generated between pH 6.8 to 7.2. In second step of ligand preparation, appropriate charges were assigned, the ligands are neutralized and then energy minimization was performed. Finally, low energy ring conformations of all the ligands were generated and then these prepared ligands were further utilized for docking study.

### Active site prediction:

Identification of small molecule binding site also used to predict the functionally important residues that helped to preserve the protein ligand interaction. The amino acid which is responsible for interaction with ligand was predicted through Sitemap module in Schrodinger 8. Using Receptor Grid Generation module in Schrodinger, the grid was generated around the active sites.

### Virtual screening:

Structure based virtual screening of compounds from chemical database is one the reliable, cost effective and time saving method for identification of new lead molecules for drug discovery. In the present study, virtual screening workflow in Schrodinger was used to screen the compounds from databases. This workflow includes three accuracy level of docking (high-throughput virtual screening [HTVS], standard precision [SP] and extra precision [XP] 9. HTVS screening was carried out using the Specs Databases, Enamics Databases, Maybridge Database and Zinc database. Funnel shaped filtering method was applied to obtained the best compounds from huge collection of compounds. At last compounds, which had good docking score, energy, h-bond interaction was selected. 

### Glide extra precision docking for the screened ligands:

All the ligands selected from the screening step were then subjected to Glide docking with extra precision (XP) to identify residues involved in hydrogen bond interactions with PBP protein. Glide XP study was carried out with default parameters. To facilitate the best possible conformation, wide range of search was carried out. Minimization cycle for Conjugate Gradient (CG) and steepest descent minimizations were used with default value of 0.05 Å for initial step size and 1.0 Å for maximum step size. In convergence criteria for the minimization, the energy change criteria and gradient criteria was set as default value of 10-7 and 0.001 kcal/mol, respectively. Following this all conformations were considered for docking studies. Glide score was used to select the best conformation for each ligand 10. Based on docking score five best ligands were chosen for Density Functional Theory (DFT) studies and further analysis 11.

### DFT:

Density Functional Theory calculations (DFT) was carried out to study the electronic features such as electron density, frontier molecular orbital density fields (i.e. HOMO, LUMO) and molecular electrostatic map. These molecular features can be used to study the biological activity and molecular properties. All DFT calculations were carried out in Schrodinger, LLC, and New York-1. Based on the solvation state the DFT calculation was carried out. Complete geometry was analyzed 12. Lowest Unoccupied Molecular Orbital (LUMO) and Highest Occupied Molecular Orbital (HOMO) energy were computed. The electrostatic potentials were calculated which provides a measure of charge distribution from the point of view of an impending reagent.

### ADME

An in silico ADME study was carried out to identify the drug likeness property of the screened compounds. Qikprop 13 module in Schrodinger was used to calculate ADME properties. Qikprop predicts the principal and physiochemical descriptors of possible drug like compounds. It also predicts the acceptability of the screened compounds, based on the Lipinski's rule of 5, which are necessary for rational drug design 14. Qikprop also compare the specific molecule properties with 95% of know drugs. It also flags 30 types of reactive functional groups that may cause false positives in high throughput screening assays. Finally, the toxicity profiles of the hits were analyzed.

### Molecular Dynamics Simulation of Docked complexes:

The binding stability of the ligand in the active site of the target and the behaviour of the protein in dynamic environment can be studied using molecular dynamics simulations (MDS) which uses an explicit solvent environment. The MD simulations were performed for the five best receptor-ligand complexes using Desmond module of Schrodinger with OPLS - AA 2005 (Optimized Potential for Liquid Simulations - All Atom) force field for minimization of the system 15. Studying the atomic level perturbation through MD simulation helps in understanding various biological aspects of molecule. These aspects include insights in structural makeup of complex or protein, conformational aspect of protein, and search of unique molecules. A protein-ligand complex was set for MD simulation stability analysis. Once the system reaches its equilibrium stage, the production run was executed. After completion, it generated various interaction diagrams, simulation trajectory, and plots. These plots were put for an analysis for checking the stability of the interaction between ligand and protein. Simulation trajectory was found to behave stably and hence it confirms the appropriate docking of ligand and protein 16.

### Chemical information:

All the chemicals and reagent used for antibacterial evaluations were obtained from Hi-media (Mumbai, India). The bacterial strains S. aureus MTCC 5021 were obtained from National Chemical Laboratory, Pune, India.

### Antibacterial activity

#### Agar disc diffusion method:

The antibacterial activity of screened compounds was tested against Staphylococcus aureus MTCC1430. The strains were collected from the microbial type culture collection, Chandigarh, India. The bacterial culture was maintained on an agar plate at 4°C and subculture for every month. The antibacterial activity was evaluated by agar well diffusion method 17. Slightly modified in brief, to prepare Muller-Hinton agar, about 3.8gm of Muller-Hinton agar was added to 100mL distilled water was autoclaved at 12°C for 15 min. The Muller-Hinton agar plates were wells cut into 6mm diameter sterile cork borer. Approximately, 10 μL of the compounds at the concentration of 10mg/mL were added into the well, incubated at room temperature for 24 hr. The effects were compared with streptomycin as a positive control.

#### Minimal Inhibitory Concentration (MIC) micro dilution broth assay method:

MIC activities of the screened compounds were analyzed by micro dilution method using resazurin indicator 18. Twofold Muller-Hinton broth was autoclaved at 121°C for 15 min. The overnight bacterial culture was grown with the final concentration of inoculum size 5 x 107 CFU/mL under the aseptic condition. The compounds were dissolved in DMSO to the concentration of 10mg/mL. The compounds were serially diluted in 96-well micro-titre plate and incubated for 18 hr at 37°C. Streptomycin for the positive and DMSO for negative control respectively. over the incubation period, 10 μL of 0.01% resazurin indicator was added and incubated for 2 hr. the microtitre plate were visible sign of growth of bacteria, the growth of bacteria changed color from blue to pink 19.

## Results and Discussion

**Figure 1:**
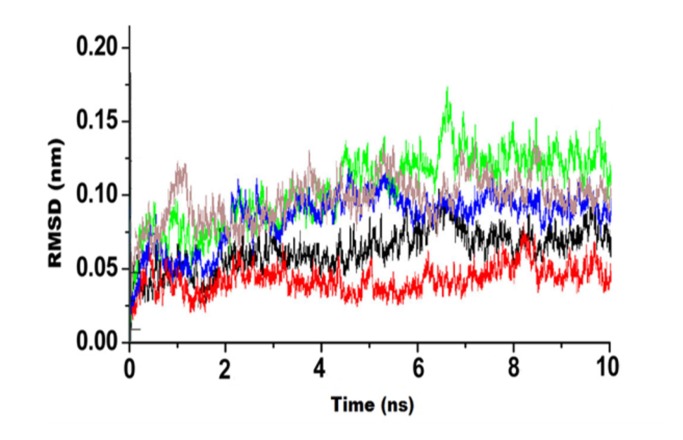
Molecular dynamics simulation of selected compounds.

**Figure 2:**
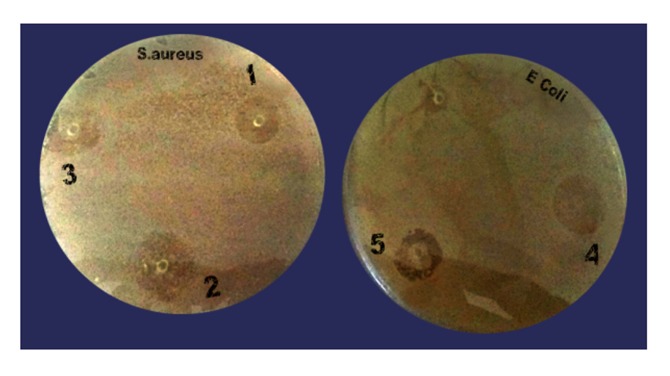
Anti-microbial activity of selected compounds.

Virtual screening against the databases search was useful resource to identify potential leads and scrutinize the inactive compounds. Analysis was done to identify the lead molecules targeting PBP protein. Phase based screening was performed against five chemical libraries and thus number of hits were obtained 20. High throughput virtual screening was carried out to identify the lead molecules. From that screening compounds, which have high scoring parameters were passed into the next level of (Standard Precision) SP docking protocol. Finally, XP docking study was carried out to obtain the better results. The docking score of the best five identified ligands from each of the five screened databases along with the glide score and glide energy with the receptor is shown in Table 1. Further Table 1 display docking score of newly identified compounds were compared with the reported one and it revealed that the former has higher docking score. 3-D structures of PBP complexed with the five identified ligands were generated. Out of five identified ligands from the screened databases, the ligand ZINC95911396 has the docking score of -10.12 k/cal. To understand the significance of newly identified compound, we have docked reported ligands 2-deoxyglucose, Lonidamine, 3-bromopyruvate, imatinib and Oxythiamine with PBP protein. The ligands have the docking score ranging from -5.88, -6.33, -7.44, -6.44 and 5.99 respectively (Table 2). Following molecular docking, the top five identified compounds were subjected to DFT studies to correlate the activity of the compounds with their electronic features. The DFT calculation investigates the electronic features of the atoms in the structure. These calculations provide the information about the global and local indices on the biological compound to their biological activity. The spatial distribution of electronic features in charge transfer mechanisms are obtained from the Highest Occupied Molecular Orbital (HOMO) and Lowest Unoccupied Molecular Orbital (LUMO). These are the better indicator for electron transport mechanism in the molecule. The electron donor and acceptor moiety of the compounds can be easily understood and this will have impact on biological function of the system. All the compounds have low HOMOLUMO energy gaps. The lower HOMO-LUMO energy gap or band gap provides higher stability of the molecule. The value of HOMO ranges from -0.21902 eV to -0.23940 eV whereas the value of LUMO ranges from -0.02989 to -0.09429 eV. MESP result reveals the location of most negative potential for enamine lies near N-methylacetamide whereas for Lifechem compound it lies near methyl-acetate. Further, most negative potential region of Maybridge is occupied by 1, 3-dimethylurea while for Zinc compound it is occupied by propan-2-one. The negative potential domain refers to the site favorable for nucleophilic attack during charge transfer reaction. Most negative potential region of SPECS correspond ethyl acetate moiety of the compound. Next, contour map analysis of frontier orbital domain reveals that HOMO orbitals for Enamine are enriched near 2, 3- dihydro -1,4 - benzo dioxine whereas HOMO orbitals of Lifechem compound lies near 1,2-dimethyl benzene. HOMO orbitals of Maybridge and Zinc compounds are distributed on N-(4-bromo-2,6- dimethylphenyl) formamide and 1-[6-hydroxy-3-methoxy-2-methoxymethyl) phenyl]ethan-1-one respectively. HOMO orbital�s of Specs compound is distributed on bis(4-chlorophenyl) (methylamino) methanol moiety of the compound. The presence of two similar electronegative groups in the proximity will discourage the catalysis of the enzyme as this will lead to electron pair repulsion. The Glu interacting with �OH of group of zinc compound but there is not electronic transfer mechanism appear for the initiate the mechanism. Also, the Lysine amino acid residues are not involved in Schiff base formation which plays a key role in the initiation of catalytic reaction and it could significantly affect the functioning of the PBP enzyme. Hence the DFT studies remains conclusive in association with frontier orbital to inhibit the biological activity of the enzyme. LUMO frontier orbital for enamine compound resides on 4-bromo-N, N-dimethyl-1Hpyrrole- 2-carboxamide whereas it occupies near 1-methyl-6-oxo- 1,6-dihydropyridine-3-carbaldehyde. Further analysis reveals that LUMO frontier orbitals for Maybridge and Zinc compounds are enriched on 1-{[3, 5-dichloro-2-methoxy-6-(methoxymethyl) phenyl] carboxyl}-3-methylurea and 1-[2-hydroxy-4-(2-hydroxy- 1-methoxypropoxy)-6-methoxyphenyl] ethan-1-one respectively. At last LUMO orbitals of specs compound is distributed on ethyl 2-(N, N'-dimethyl hydrazine carbonyl) benzoate domain of the compound. Band gap of HOMO-LUMO energy gap signifies the stability and chemical reactivity of the complex. Lower band gap facilitates chemical reactivity whereas higher band gap renders stability to the complex and consequently decreases chemical reactivity. As the value of band gap for ZINC database compound is comparatively lower -0.1906 eV, it favors more the reactivity and stability of the complex. Removable of electron from frontier orbital (HOMO) will be lower than the higher energy gap, so energy absolute value will have good inhibitory effect. In this study, we have assessed the top 5 compounds from different databases to check the drug likeliness and pharmaceutical relevant properties such as pharmacokinetics consist of ADME. The QikProp module implemented in the Schrodinger software suite was estimated the drug likeliness of the compound. The calculated ADME properties for the top five compounds were given in Table 2. The top 5 compounds of Molecular weight (130.0-725.0), QP log P (o/w) ( -2:0 to 6.5), QPPCaco (25 is poor and > 500 is great), IC 50 value for blockage of HERG K+ channels (concern below -5:0), LogP MDCK, percentage of human oral absorption were predicted in the acceptable range which provokes the drug ability of the compound. Calculated ADME properties of the compounds were shown in Table 3. The initial 5 docked complexes of PBP protein were subjected to MDS studies for analyzing the stability in terms of RMSD (Root Mean Square Deviation) and the potential interactions for the inhibition of the molecule was identified during 10 ns time periods. The RMSD plots of the five complexes are collectively shown in Figure 1. Moreover, it is necessary to understand the interaction of docked complexes during 30 ns time periods for inhibiting mechanism. In term of PBP-Specs database complex, the backbone RMS deviation values were found in the average range of 0.1-0.25 nm and in the initial 2500 ps time period, the complex has highly stable in the range of 0.1-0.15 followed that deviation values are increasing up 0.3 nm and attain stable conformation throughout 10 ns time period. In term of PBP- Enamics complex, the backbone RMSD values found between in the average range of 0.1-0.25 nm and the values increasing since the initial time period to final 10 ns. In the complex of PBP- Maybridge, the backbone RMSD values found in the average range between 01.03 nm in the initial 23 ns time periods and the remaining deviation values were highly stable in 10 ns time period. In the complex of PBP-TOSLAB, the backbone RMS deviation values were found in the average range of 0.1-0.25 nm. In term of PBP-Zinc complexes, the backbone RMS deviation values were found in the average range of 0.1-0.25 nm and in the initial 2500 ps time period, the complex has highly stable in the range of 0.1-0.15 followed that deviation values are increasing up 0.3 nm and attain stable conformation throughout 10 ns time period. In vitro anti-microbial activities of best five compounds were analyzed through antibacterial activity test (Figure 2). Obtained results clearly informed us, the selected compounds from virtual screening showed very good activity against E.coli and S. aureus. Out of five compounds, compound from ZINC database (ZINC95911396) the good zone of clearance. A result of invitro study was shown in Table 4. The antimicrobial activity screened compounds showed significant zone of inhibition. In addition MIC readings are also noteworthy (Table 5). O'Donnell et al. 21 reported that compounds have very strong biological activity at < 10 μg/ml. Our results were similar to the currently used antibiotic against S. aureus 22,23[22, 23]. Identification of new drugs for S. aureus infection is one of important field in antimicrobial related research study. Hence our present research provided the compounds that can be explored for further therapeutic agents against S. aureus infection.

## Conclusion

PBP was targeted using rational approach with predictive ability for inhibiting S. aureus. Comparative docking studies were performed for screened compounds and known compounds. The screened compounds have chemical features similar to reported compounds. Molecular docking analysis of the screened compounds revealed the catalytic residues play a vital role in inhibiting the biological activity of PBP. Results show that the HOMO (nucleophilic) regions of the identified compounds are interacting with these residues facilitating inhibition of the activity of PBP protein. The stability of the docked protein-ligand complexes was confirmed using (Molecular dynamics simulation) MDS studies of docked compound with PBP. Interactions with the target and electronic features of the screened compounds are in the design of novel inhibitors of PBP of S. aureus.

## Figures and Tables

**Table 1:** Glide score, glide energy of selected from virtual screening compounds

S. No	Compound ID	Docking Score	Glide Energy	Glide e model
1	742503(Specs)	-9.411	87.544	-80.27
2	742505(Enamics database)	-8.002	73.452	-80.27
3	00007(Maybridge c database)	-9.196	89.828	-79.958
4	95911396 (ZINC Database)	-10.12	94.46	-90.163
5	00004(TOSLAB)	-8.546	77.421	-75.159

**Table 2:** Glide score and glide energy of already reported ligands

S. No	Compound id	Glide score	Glide energy
1	2-deoxyglucose	-5.88	45.76
2	Lonidamine	-6.33	52.82
3	3-bromopyruvate	-7.44	63.18
4	Imatinib	-6.44	58.48
5	Oxythiamine	-5.99	51.08

**Table 3:** Predicted ADME properties of selected compounds through Qikprop analysis

S. No.	Compound id	Molecular weight (g per mol)a	QP log P (o by w)b	QPPCacoc	QPLog HERG d	LogPMDCKe	Percentage of Human oral absorptionf
1	742503 (Specs database)	157.65	3.9	391.11	-6.781	124.3	95.61
2	742505 (Enamics)	476.81	4.87	535.24	-6.879	152.216	67.485
3	00007 (Maybridge database)	432.76	5.23	579.34	-5.853	159.289	66.815
4	95911396 (ZINC Database)	321.23	4.24	650.29	-6.234	168.578	85.698
5	00004(TOSLAB)	589.54	5.17	432.42	-5.867	162.578	80.141

aMolecular weight of the molecule. (Acceptable range 130.0-725.0.); bPredicted octanol/water partition coeffcient log P (acceptable range 2:0 to 6.5); cPredicted Caco-2 cell permeability in nm/s (acceptable range: less than 25 is poor and greater than 500 is great); dPredicted IC 50 value for blockage of HERG K+ channels (concern below -5:0); ePredicted apparent MDCK cell permeability in nm/s; fPercentage of human oral absorption (acceptable range: less than 25 percent is poor and greater than 80 percent is high)							

**Table 4:** Antibacterial activity of selected compounds

S. No	Compound Id	Diameter of inhibition zone (cm)
1	742503 (Specs database)	1.6
2	742505 (Enamics)	1.8
3	00007 (Maybridge database)	1.4
4	95911396 (ZINC Database)	1.8
5	00004 (TOSLAB)	1.5

**Table 5:** MIC value of the Screened compounds

S. No	Compound Id	MIC (micro g per ml)
1	742503 (Specs database)	9
2	742505 (Enamics)	7
3	00007 (Maybridge database)	8
4	95911396 (ZINC Database)	2
5	00004(TOSLAB)	4
